# Development and validation of a combined diagnostic model for prostate cancer integrating MRI parameters with p504s, CK5/6, and Ki-67 expression

**DOI:** 10.1515/biol-2025-1244

**Published:** 2026-01-23

**Authors:** Qingchang Ren, Jialong Gu, Nankang Lu

**Affiliations:** Department of Radiology, The Second People’s Hospital, Wuhu, Wuhu, 241000, Anhui, China; Huashan Hospital Fudan University, Shanghai, 200040, Shanghai, China; Department of Radiology, The First Affiliated Hospital of Hainan Medical College, Haikou, 570102, Hainan, China

**Keywords:** prostate cancer, MRI parameters, *α*-Methylacyl-CoA racemase, cytokeratin 5/6, Ki-67

## Abstract

This study aimed to develop and validate a diagnostic model for prostate cancer (PCa) by integrating magnetic resonance imaging (MRI) parameters with the immunohistochemical expression of p504s, CK5/6, and Ki-67. A total of 448 patients undergoing prostate needle biopsy were included and randomly allocated into training (70 %) and validation (30 %) cohorts. Clinical data, MRI findings, and biomarker expression levels were analyzed. Multivariate logistic regression identified independent predictors, which were used to construct a diagnostic nomogram. Compared to controls, PCa patients had significantly higher PSA levels, lower f-PSA/t-PSA ratios, a greater frequency of palpable nodules, higher CC/C ratios, lower ADC values, increased p504s and Ki-67 positivity, and reduced CK5/6 expression. Seven variables were ultimately identified as independent predictors for the model. The resulting nomogram demonstrated excellent discrimination, with an area under the curve (AUC) of 0.971 in the training set and 0.977 in the validation set. It significantly outperformed a model using clinical indicators alone. This combined MRI-biomarker model shows high diagnostic accuracy for PCa and could potentially aid clinical decision-making and reduce unnecessary biopsies. External validation is required prior to clinical application.

## 1 Introduction

Prostate cancer (PCa) is one of the most common cancers in men worldwide, with incidence increasing with age [1]. Early diagnosis and intervention are critical for improving patient outcomes. Current diagnostic modalities, including prostate-specific antigen (PSA) testing, digital rectal examination (DRE), and prostate needle biopsy, are widely used but have limitations. For instance, PSA testing lacks sufficient specificity and offers limited utility for subsequent disease evaluation and therapeutic guidance. Consequently, the exploration of more effective assessment methods to inform clinical decision-making remains an ongoing need [2].

Recent advancements in imaging technologies have established the diagnostic value of magnetic resonance imaging (MRI) in PCa [3], 4]. Quantitative parameters from MRI, such as the apparent diffusion coefficient (ADC) and dynamic contrast-enhanced (DCE)-MRI time-intensity curves, capture microstructural and functional changes in prostatic tissues and contribute to oncological assessment. Nevertheless, the standalone diagnostic performance of these metrics requires further optimization [5], 6], necessitating integrative analysis combining MRI-derived parameters with complementary biomarkers.

Notably, *α*-methylacyl-CoA racemase (AMACR, also known as p504s) has emerged as a well-established diagnostic biomarker for PCa. Cytokeratin 5/6 (CK5/6), a cytoskeletal protein, exhibits expression patterns correlating with tumor aggressiveness and prognosis [7]. Ki-67, a nuclear proliferation antigen, serves as a robust indicator of cellular proliferative activity, enabling stratification of malignant potential and prognostic evaluation [8], 9].

Therefore, this study aims to develop and validate a composite diagnostic model that integrates MRI parameters with immunohistochemical (IHC) expression levels of p504s, CK5/6, and Ki-67. Unlike previous studies focusing primarily on MRI features or single biomarkers, our approach integrates multiple IHC indicators with quantitative MRI parameters to improve diagnostic accuracy and clinical decision-making. This integrated model may provide a more comprehensive and individualized assessment for patients with suspected PCa.

## 2 Materials and methods

### 2.1 General data

A retrospective cohort study was conducted on patients undergoing prostate needle biopsy at our hospital between August 2020 and August 2024. Participants were stratified into two groups based on histopathological findings: a PCa group (case group, *n* = 120) and a non-PCa group (control group, *n* = 328). Sample size determination was based on the events per variable (EPV) criterion for regression modeling, considering MRI parameters and 3 molecular biomarkers. With an anticipated inclusion of approximately 10 predictor variables in the final regression analysis, the minimum required case number was estimated at 100–200 (10–20 EPV) according to established methodological literature [10]. Our total sample size of 448 participants (120 cases; 328 controls) exceeded this threshold and maintained a case-to-control ratio of 1:2.7, consistent with conventional case-control study design parameters. The total of 120 cases met the minimum requirement of 10 × 10 = 100 cases. The cohort was randomly partitioned into training (*n* = 314, 70 %) and validation (*n* = 134, 30 %) subsets.

Inclusion criteria comprised: (1) age ≥50 years; (2) clinical suspicion of PCa necessitating biopsy (manifesting ≥1 of: abnormal DRE, elevated PSA, or suspicious imaging findings); (3) completion of preoperative multiparametric MRI (mpMRI); (4) availability of definitive pathological diagnosis from biopsy specimens; (5) accessible IHC results for p504s, CK5/6, and Ki-67; and (6) comprehensive clinical documentation.

Exclusion criteria encompassed: (1) prior PCa-directed therapies (surgery, radiotherapy, or androgen deprivation); (2) contraindications to MRI; (3) suboptimal pathological specimen quality precluding analysis; (4) concurrent active malignancies; or (5) insufficient clinical data for robust analysis.

**Informed consent**: Informed consent has been obtained from all individuals included in this study.

**Ethical approval**: The research related to human use has been complied with all the relevant national regulations, institutional policies and in accordance with the tenets of the Helsinki Declaration, and has been approved by the Human Ethics Committee of The Second People’s Hospital, Wuhu.

## 3 Methods

### 3.1 Data collection

Demographic and clinical parameters were obtained from the electronic medical record (EMR) system of our hospital. Documented data included patient age, body mass index (BMI), PSA, total PSA (tPSA), free PSA (fPSA), DRE findings, hypertension, diabetes mellitus, tobacco use, and alcohol consumption. All patient data were de-identified prior to analysis, and no personal identifiers were accessible to the investigators, in compliance with institutional and Health Insurance Portability and Accountability Act (HIPAA) privacy standards.

### 3.2 MRI parameter acquisition

Diffusion-weighted imaging (DWI) and proton magnetic resonance spectroscopy (^1^H-MRS) sequences were acquired using a Siemens 3.0T superconducting MRI scanner with an 18-channel phased-array body coil.

For DWI, the parameters were as follows: repetition time/echo time (TR/TE) = 4,400/85 ms, slice thickness/gap = 4/0.8 mm, matrix size = 192 × 85 %, field of view (FOV) = 28 × 28 cm^2^, and acquisition time of 1 min 50 s. Image data were transferred to a Siemens Syngo MR B19 workstation. Within the Viewing interface, a radiologist selected the slice demonstrating the largest lesion cross-section and measured the ADC value within the suspicious lesion.

The ^1^H-MRS sequence utilized the following parameters: TR/TE = 940/145 ms, FOV = 12 × 12 cm^2^, and acquisition time of 10 min 12 s. Prior to MRS scanning, localization was performed using coronal, sagittal, and axial T2-weighted imaging (T2WI) sequences to ensure complete prostate gland coverage. Standard practice included the application of 8 saturation bands around the volume of interest to minimize motion and chemical shift artifacts. Manual shimming was performed immediately before data acquisition to optimize field homogeneity and spectral quality. After scanning, spectroscopic data were transferred to the Siemens Syngo MR B19 workstation. Automated post-processing was conducted using the workstation’s dedicated MRS analysis software, which analyzed spectral peaks for choline (Cho), creatine (Cr), and citrate (Cit), and calculated the (Cho + Cr)/Cit ratio (CC/C ratio).

### 3.3 Detection of p504s, CK5/6, and Ki-67

**Figure 1:**
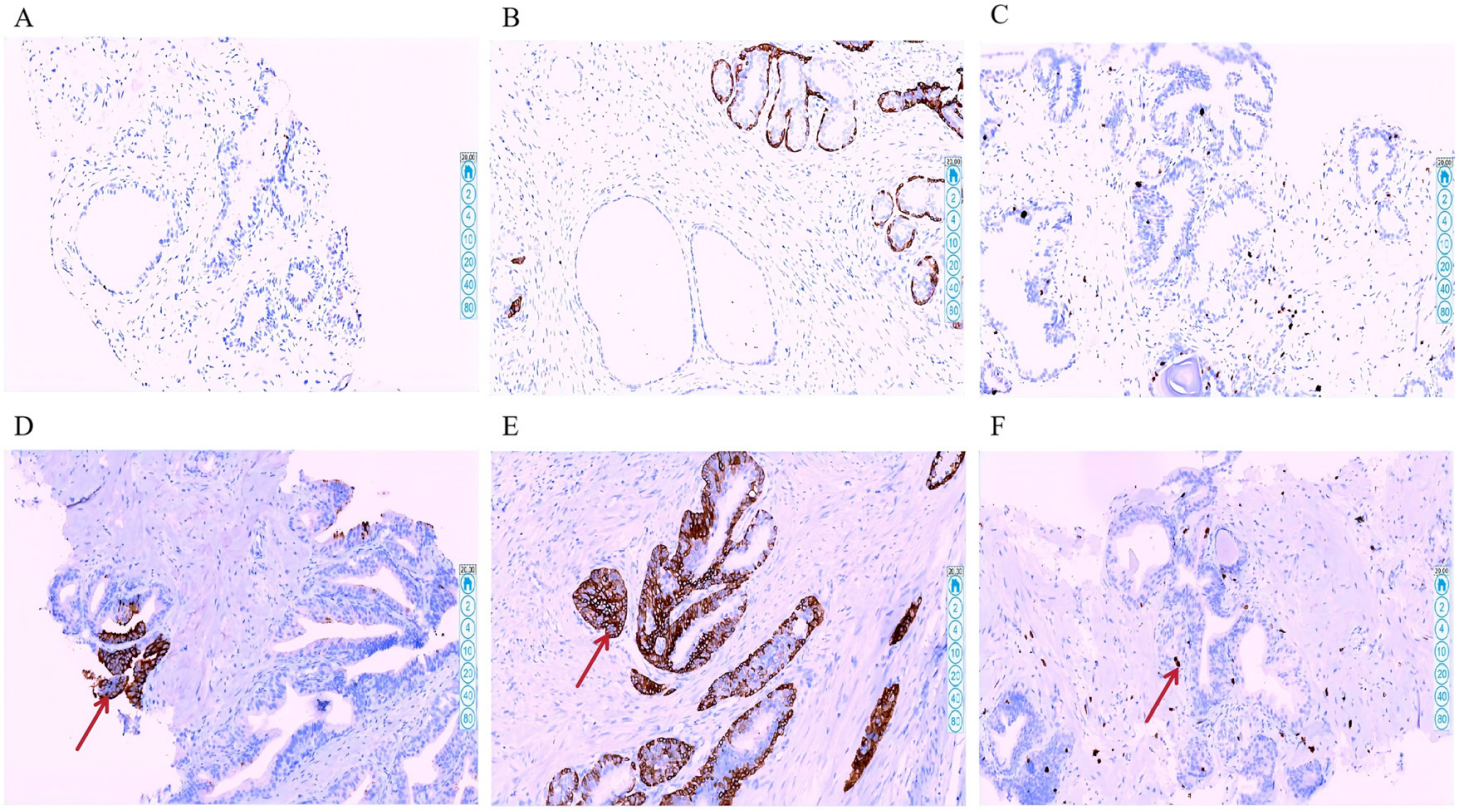
Immunohistochemical staining of p504s, CK5/6, and Ki-67 in negative and positive cases. Note: Representative immunohistochemical staining images of p504s, CK5/6, and Ki-67 in tissue sections. Panels A–C show negative expression for p504s (A), CK5/6 (B), and Ki-67 (C), respectively. Panels D–F demonstrate positive staining for p504s (D), CK5/6 (E), and Ki-67 (F), with red arrows indicating regions of positive immunoreactivity.

Mouse anti-human monoclonal antibodies against p504s, CK5/6, and Ki-67 were purchased from Zhongshan Golden Bridge Biotechnology Co., Ltd. (Beijing, China). Expression levels in tissue samples were evaluated using IHC (Figure 1). For interpretation, a pathologist randomly examined 10 high-power fields. Positive immunoreactivity was defined as distinct brownish-yellow granular staining significantly above background levels, localized to the cytoplasm for p504s and CK5/6, and to the nucleus for Ki-67. Staining was semi-quantitatively assessed using a combined score derived from staining intensity and the proportion of positive cells (total score range: 0–9), with a total score of ≥2 considered positive. Staining intensity was scored as follows: 0 (no staining), 1+ (weak, light yellow), 2+ (moderate, brownish-yellow), and 3+ (strong, dark brownish-yellow). The proportion of positive cells was categorized as: 0 (<5 %), 1 (5–25 %), 2 (26–50 %), and 3 (>50 %). The total score was calculated by multiplying the intensity score by the proportion score.

### 3.4 Statistical analysis

All statistical analyses were performed using SPSS software (version 22.0). Cases with missing MRI or biomarker data were excluded, and no imputation was performed. Categorical variables, including the presence of nodules on DRE, hypertension, diabetes mellitus, tobacco use, and alcohol consumption, were presented as frequencies and percentages and compared using the chi-square (χ^2^) test. Continuous variables that conformed to a normal distribution, such as age, BMI, tPSA, and fPSA, were expressed as mean ± standard deviation ( $\bar{x}$ ± s) and analyzed using *t*-tests. Logistic regression analysis was employed to identify significant influencing factors. A nomogram prediction model was constructed using R software. Model performance was evaluated using receiver operating characteristic (ROC) curve analysis, calibration curves, and decision curve analysis (DCA). A *P*-value < 0.05 was considered statistically significant.

## 4 Results

### 4.1 Univariate analysis of PCa occurrence

Compared to the control group, the case group exhibited significantly higher PSA levels, lower f-PSA/t-PSA ratios, a greater prevalence of palpable nodules on DRE, higher CC/C ratios, lower ADC values, higher p504s and Ki-67 positivity, and lower CK5/6 expression (Table 1, all *P* < 0.05).

**Table 1:** Patient baseline characteristics and univariate analysis of prostate cancer occurrence ( $\bar{x}$ ± s)/M (Q_25_, Q_75_).

Parameters		Case group (*n* = 120)	Control group (*n* = 328)	*t/χ* ^2^/Z	*P*
Age (years)		57.52 ± 3.84	56.98 ± 3.87	1.314	0.189
BMI (kg/m^2^)		22.01 ± 1.75	22.12 ± 1.89	−0.556	0.578
PSA (ng/mL)		12.22(9.42, 14.94)	8.89(6.72, 11.47)	−8.327	<0.001
f-PSA/t-PSA ratio		0.17(0.11, 0.21)	0.22(0.18, 0.28)	−8.406	<0.001
DRE nodules	Present	70(58.33)	102(31.10)	27.553	<0.001
	Absent	50(41.67)	226(68.90)		
Hypertension	Present	32(26.67)	108(32.90)	1.603	0.206
	Absent	88(73.33)	220(67.07)		
Diabetes mellitus	Present	21(17.50)	60(18.29)	0.037	0.847
	Absent	99(82.50)	268(81.71)		
Smoking history	Present	98(81.67)	270(82.32)	0.025	0.874
	Absent	22(18.33)	58(17.68)		
Alcohol consumption history	Present	80(66.67)	228(69.51)	0.331	0.565
	Absent	40(33.33)	100(30.49)		
ADC value (× 10^−3^ mm^2^/s)		0.59(0.45, 0.73)	0.92(0.65, 1.17)	−8.922	<0.001
CC/C ratio		1.59(0.54, 3.64)	0.88(0.34, 1.52)	−5.449	<0.001
p504s	Positive	41(34.17)	30(9.15)	41.240	<0.001
	Negative	79(65.83)	298(90.85)		
CK5/6	Positive	31(25.83)	279(85.06)	144.591	<0.001
	Negative	89(74.17)	49(14.94)		
Ki-67	Positive	65(54.17)	65(19.82)	50.328	<0.001
	Negative	55(45.83)	263(80.18)		

### 4.2 Multivariate analysis of PCa occurrence

Variables demonstrating significant differences in univariate analysis were included in a multivariate logistic regression analysis, with the coding scheme detailed in Table 2. The results identified p504s, Ki-67, CK5/6, PSA, fPSA/tPSA ratio, CC/C ratio, and ADC value as independent risk factors for PCa (all *P* < 0.05), as shown in Table 3.

**Table 2:** Variable coding scheme.

Variable type	Variable name	Value assignment
Dependent	Group	1 = Case group, 0 = Control group
Independent	DRE nodules	1 = Present, 0 = Absent
	p504s	1 = Positive, 0 = Negative
	CK5/6	1 = Positive, 0 = Negative
	Ki-67	1 = Positive, 0 = Negative
	PSA	Continuous variable
	f-PSA/t-PSA	Continuous variable
	ADC value	Continuous variable
	CC/C	Continuous variable

**Table 3:** Multivariate logistic regression analysis of prostate cancer occurrence.

Variable	B	SE	Wald	df	*P*	OR	95 % CI for EXP(B)	
							Lower	Upper
DRE nodules (1)	0.569	0.511	1.241	1	0.265	1.767	0.649	4.811
p504s (1)	2.770	0.711	15.181	1	<0.001	15.965	3.962	64.328
CK5/6 (1)	−2.128	0.537	15.695	1	<0.001	0.119	0.042	0.341
Ki-67 (1)	1.860	0.581	10.242	1	0.001	6.423	2.056	20.063
PSA	1.312	0.252	27.060	1	<0.001	3.715	2.266	6.092
f-PSA/t-PSA ratio	−34.000	7.359	21.344	1	<0.001	<0.001	<0.001	<0.001
ADC value	−7.920	1.626	23.731	1	<0.001	<0.001	<0.001	0.009
CC/C ratio	1.433	0.344	17.378	1	<0.001	4.190	2.136	8.218
Constant	−5.036	1.389	13.144	1	<0.001	0.006		

### 4.3 Development of a PCa diagnostic model incorporating MRI parameters, p504s, CK5/6, and Ki-67

**Figure 2:**
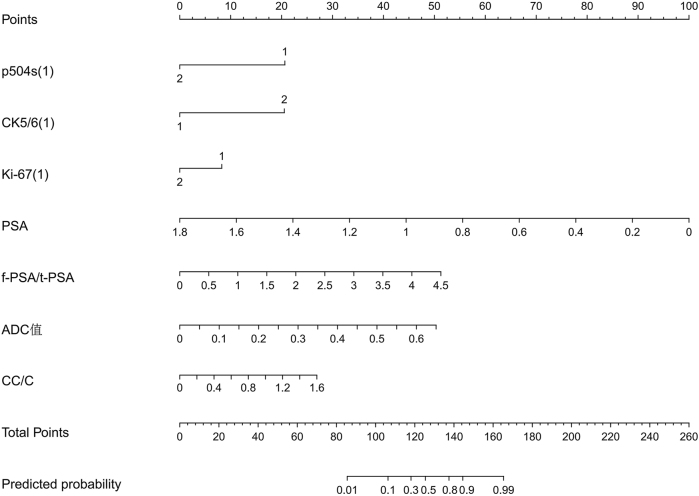
Nomogram for prostate cancer diagnosis based on MRI parameters, p504s, CK5/6, and Ki-67. Note: Each variable is assigned points according to its contribution in the multivariate logistic regression model, as shown on the upper “points” scale. For an individual patient, locate each variable’s value, including p504s (1), CK5/6 (1), Ki-67 (1), PSA, f-PSA/t-PSA, ADC value, and CC/C, draw a vertical line to determine its score, and sum all points to obtain the “total points”. Projecting this total downward to the “predicted probability” axis estimates the likelihood of prostate cancer. Here, p504s (1), CK5/6 (1), and Ki-67 (1) indicate immunohistochemical expression levels (1 = low/negative, 2 = high/positive); PSA is prostate-specific antigen; f-PSA/t-PSA is the free-to-total PSA ratio; ADC value is derived from diffusion-weighted MRI; and CC/C is the (choline + creatine)/citrate ratio from proton magnetic resonance spectroscopy. Higher total points correspond to a greater probability of prostate cancer, helping clinicians assess risk and guide biopsy decisions.

Based on the multivariate logistic regression results, a diagnostic model for PCa was developed, incorporating the identified independent risk factors: MRI parameters, p504s, CK5/6, and Ki-67. This model is visualized in Figure 2.

### 4.4 Validation of the PCa diagnostic model incorporating MRI parameters, p504s, CK5/6, and Ki-67

**Figure 3:**
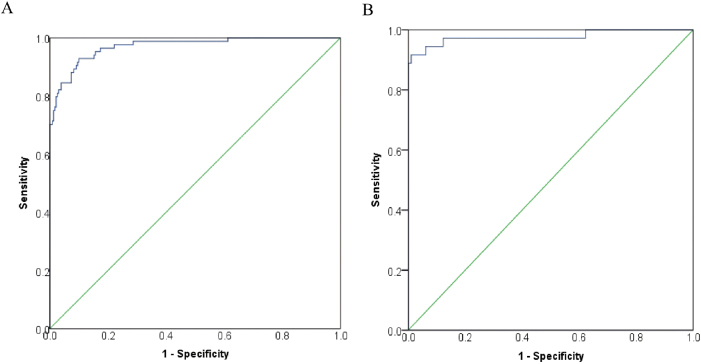
ROC curves for the diagnostic model in the training and validation sets. Note: A, training set; B, validation set. The *x*-axis represents 1-specificity and the *y*-axis represents sensitivity. The diagonal green line indicates the reference line of random prediction. The model shows high area under the curve (AUC) values in both datasets, demonstrating excellent discriminatory performance between prostate cancer and benign lesions.

**Figure 4:**
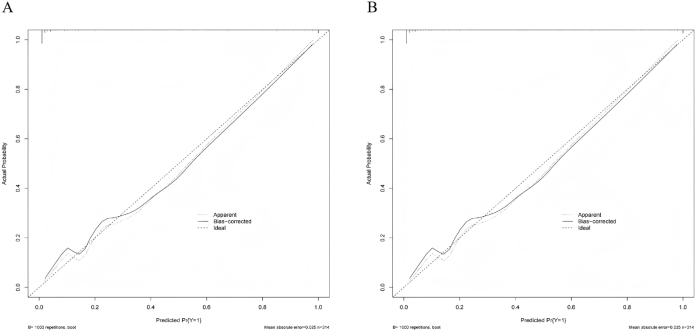
Calibration curves for the diagnostic model in the training and validation sets. Note: A, training set; B, validation set. The *x*-axis represents the predicted probability of prostate cancer, and the *y*-axis represents the observed probability. The dashed line (“ideal”) indicates perfect prediction, the solid line (“apparent”) represents the model’s performance on the original dataset, and the dotted line (“bias-corrected”) shows the performance after bootstrap correction. The close alignment between the curves and the ideal line demonstrates good calibration of the model, with minor fluctuations at low probability ranges likely due to fewer cases in that interval.

**Figure 5:**
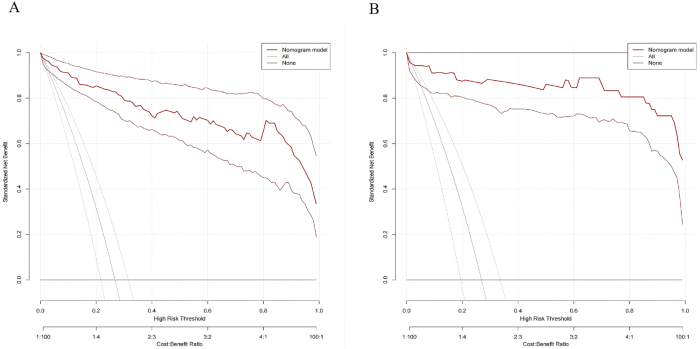
Decision curves for the diagnostic model in the training and validation sets. Note: A, training set; B, validation set. The *x*-axis represents the threshold probability of prostate cancer, and the *y*-axis represents the standardized net benefit. The red curve denotes the nomogram model, while the gray lines represent the “treat-all” and “treat-none” strategies. The nomogram model provides a higher net clinical benefit across most threshold probabilities in both cohorts, indicating superior clinical utility for guiding biopsy decisions.

The discriminatory ability of the model was assessed using ROC curve analysis. It demonstrated excellent discrimination in both the training set [area under the curve (AUC) = 0.971; 95 % *confidence interval* (*CI*): 0.952–0.990] and the validation set (AUC = 0.977; 95 % CI: 0.943–1.000) (Figure 3). Model calibration, evaluated via calibration curves, revealed minimal deviation from the ideal reference line in both sets, indicating close agreement between predicted probabilities and actual outcomes (Figure 4). Minor fluctuations in the very low-probability range likely reflect limited sample counts there. DCA, plotting net benefit against the threshold probability for defining high-risk status, demonstrated the model’s clinical utility. The decision curves for both datasets lay above the “treat none” and “treat all” reference lines across a clinically relevant range of threshold probabilities, indicating superior net benefit compared to alternative strategies (Figure 5).

### 4.5 Comparison of the nomogram model with a clinical prediction model

**Figure 6:**
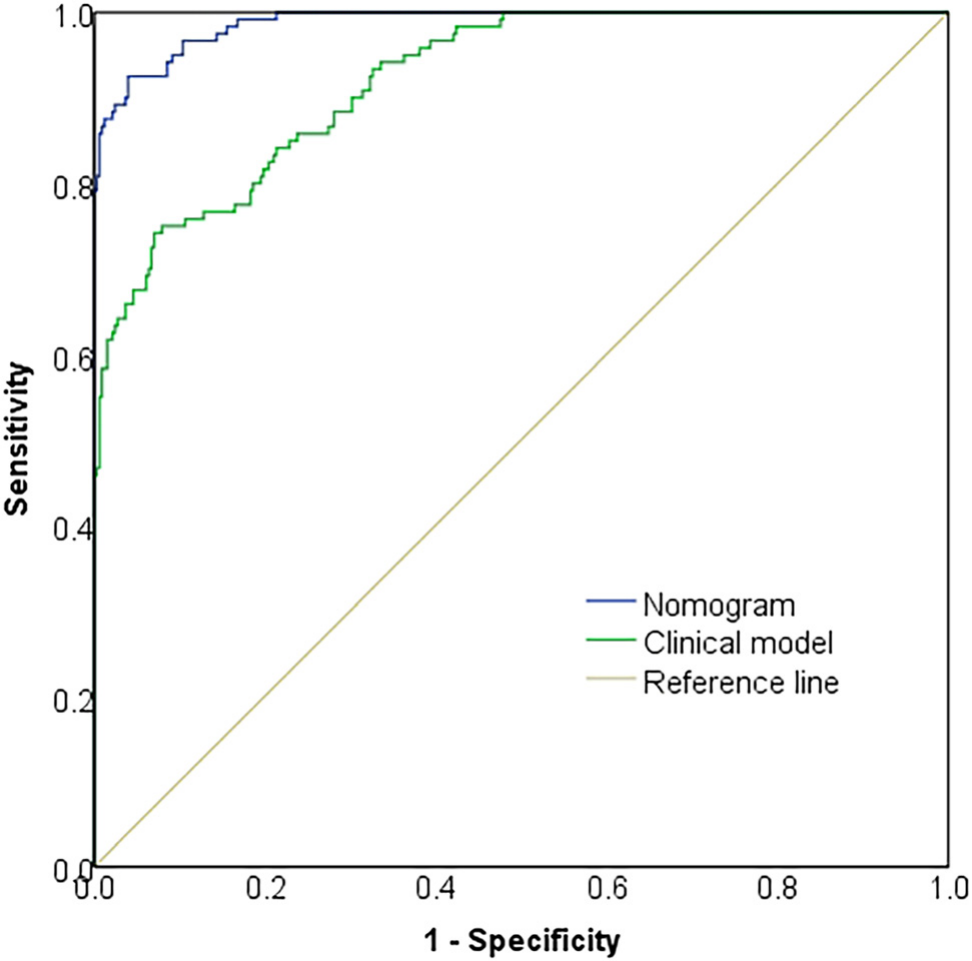
Comparison of the nomogram-based diagnostic model and the clinical prediction model. Note: The blue line represents the nomogram model, the green line represents the clinical model based on conventional clinical parameters, and the diagonal yellow line indicates the reference line for random prediction. The nomogram model demonstrates a higher area under the curve (AUC) than the clinical model, indicating superior discriminative ability and improved diagnostic accuracy.

Using PCa diagnosis as the endpoint, a clinical prediction model was constructed incorporating variables selected through multivariate logistic regression analysis (DRE nodules, PSA levels, and f-PSA/t-PSA ratio). Compared to this clinical model, the nomogram-based diagnostic model integrating MRI parameters, p504s, CK5/6, and Ki-67 demonstrated significantly superior discriminative performance. The nomogram model achieved an AUC of 0.988 (95 % *CI*: 0.981–0.995), significantly exceeding the clinical model’s AUC of 0.921 (95 % *CI*: 0.895–0.948), as displayed in Figure 6.

## 5 Discussion

PCa is a prevalent malignancy in men globally, where accurate early-stage diagnosis is critical for guiding therapy and improving survival [11]. Recent advancements have increased the application of MRI in PCa screening and evaluation. However, as Fazekas et al. [12] highlighted, while MRI enhances tumor detection, its standalone application provides insufficient information on tumor cell characteristics and disease progression, limiting its utility for guiding definitive treatment. In contrast, tissue-based pathological markers offer a multidimensional scientific foundation for evaluating treatment options by precisely delineating tumor molecular characteristics. Biomarkers such as p504s, CK5/6, and Ki-67 exhibit a well-established correlation with PCa aggressiveness. Supporting this, research by Yoshizawa et al. [13] underscores the value of p504s and Ki-67 as significant diagnostic and prognostic indicators. Consequently, this study integrated MRI-derived parameters with these key gene expression levels to construct a combined diagnostic model.

Our analysis revealed significant differences between the case and control groups. Cases demonstrated elevated PSA levels, reduced f-PSA/t-PSA ratios, and a higher prevalence of palpable nodules on DRE. These reflect established biological hallmarks of PCa: elevated PSA is a primary screening indicator, a diminished f-PSA/t-PSA ratio increases the probability of malignancy, and palpable nodules are a frequent clinical sign. Beyond these, MRI parameters further distinguished the groups, with cases exhibiting lower ADC values and higher CC/C ratios. These observations are consistent with the findings of Fang et al. [14], who confirmed that reduced ADC values correlate with densely packed tumor cells, while elevated CC/C ratios associate with tumor angiogenesis. ADC quantifies water molecule diffusion within tissue, and lower values typically indicate high cellular density or intracellular edema, which are features common in PCa. Conversely, alterations in the CC/C ratio reflect the metabolic state of prostatic tissue, with higher ratios suggesting probable neoplastic tissue.

Significant disparities were also observed in biomarker expression. Cases had higher positive staining for p504s and Ki-67 and lower for CK5/6. Elevated p504s and Ki-67 expression links to heightened cellular proliferation and metabolic activity, characteristic of PCa. Furthermore, diminished CK5/6 expression validates the research of Iakymenko et al. [15], which identified CK5/6 loss in basal cells as an early event in carcinogenesis. This reduced expression likely reflects tumor cell dedifferentiation, where malignant cells typically show diminished CK5/6 levels. Recent studies reinforce the clinical relevance of our biomarker panel. Ki-67 has been reported to associate with adverse outcomes and aid diagnostic and prognostic assessment in PCa cohorts, supporting its use alongside routine markers. In practice, basal-cell stains such as CK5/6 (often within PIN-4 panels) help delineate benign glands by highlighting basal cells, thereby complementing positive markers and improving diagnostic confidence in challenging foci. These observations align with our choice to pair MRI parameters with readily available IHC markers to enhance interpretability and scalability [16]. Other molecular markers such as PTEN and ERG were not included, as these tests are not routinely performed in our institution and were unavailable for sufficient cases in this retrospective cohort.

Multivariate logistic regression analysis further identified p504s, Ki-67, CK5/6, PSA, f-PSA/t-PSA ratio, CC/C ratio, and ADC value as independent risk factors for PCa. This underscores the multidimensional nature of PCa pathogenesis. PSA and f-PSA/t-PSA ratio, as classic serum biomarkers, provide foundational screening information. Elevated PSA levels raise suspicion, while a reduced f-PSA/t-PSA ratio increases malignancy probability, mechanistically linked to tumor-induced alterations in PSA molecular forms. MRI parameters, CC/C ratio and ADC value, deliver complementary information on tumor metabolism and tissue microstructure. Our results are consistent with contemporary evidence that integrating mpMRI with clinical or biomarker information can improve discrimination beyond clinical variables alone. Recent nomogram and risk-calculator studies have shown that MRI-inclusive or multimodal models achieve higher AUCs and can help reduce unnecessary biopsies compared with clinical-only approaches, which supports the pragmatic value of our MRI-biomarker nomogram [17].

The CC/C ratio, derived from MRS, reflects the signal intensity contrast between tumor and adjacent normal tissue, aiding boundary delineation. A core pathological feature of PCa involves the abnormal proliferation and disorganized arrangement of tumor cells, forming dense nests or glandular structures. When cellular density markedly exceeds normal prostatic tissue, characteristic alterations occur in water molecule distribution, extracellular space, and vascular architecture. On MRI, resultant reduced free water in dense tumor creates signal intensity differences compared to normal glandular and stromal tissue, manifesting as an elevated CC/C ratio. Furthermore, if tumor cells compress surrounding capillaries, altering local hemodynamics, contrast agent distribution and washout patterns differ from normal tissue. Post-contrast enhancement typically yields significantly higher signal intensity within the tumor, further contributing to an increased CC/C value [18]. This contrast maps the inherent tumor tissue heterogeneity onto the image, with cellular density and architectural disarray representing key features distinguishing carcinoma from benign hyperplasia. The ADC value quantifies water molecule diffusion restriction. The integrity and functionality of cancer cell membranes often differ from normal cells, potentially exhibiting aberrant membrane transporter expression, dysfunctional ion channels, and reduced membrane fluidity. These alterations can impede water molecule diffusion efficiency across membranes [19]. Concurrently, the heightened metabolic state of PCa cells elevates intracellular osmotic pressure, promoting water influx into cells. This further depletes extracellular water content, contributing to reduced ADC values.

The biomarkers p504s, Ki-67, and CK5/6 illuminate distinct dimensions of tumor biology, such as metabolic dysregulation, proliferative activity, and cellular differentiation status, respectively. p504s, a key enzyme in fatty acid metabolism, is scarcely expressed in normal prostate tissue but markedly upregulated in PCa cells. To fuel rapid proliferation, cancer cells necessitate substantial energy, prompting metabolic reprogramming. This includes upregulating p504s activity to remodel fatty acid *β*-oxidation pathways, generating acetyl-CoA which is a crucial substrate for energy production and biosynthetic processes [20]. Additionally, p504s modulates lipid peroxidation levels, mitigating intracellular reactive oxygen species (ROS) accumulation, thereby aiding cancer cells in evading oxidative stress-induced death. Consequently, p504s positivity directly signifies underlying metabolic aberrations and is a significant molecular hallmark of prostate carcinogenesis. Ki-67, a nuclear antigen associated with cellular proliferation, is expressed exclusively during active phases of the cell cycle. Its labeling index provides a direct measure of tumor cell proliferative activity [21]. Corroborating its clinical relevance, Jha et al. [22] demonstrated a significant positive correlation between Ki-67 expression levels and PCa Gleason scores. CK5/6 serves as a specific marker for basal cells in prostate acini, playing a vital role in maintaining glandular structural integrity and cellular differentiation. The loss of CK5/6 immunoreactivity signifies that malignant cells have breached the basal cell layer, acquiring the capacity for stromal invasion. This transition is a critical juncture in the progression from prostatic intraepithelial neoplasia to invasive carcinoma, as CK5/6 absence associates with enhanced migratory and invasive potential in cancer cells.

The developed and validated nomogram model demonstrated high discriminative ability and good calibration. Calibration curves for both training and validation sets exhibited high concordance with the ideal reference line, indicating remarkable alignment between the model’s predicted probabilities and actual observed outcomes, though slight instability occurred in the low-probability region. This pattern likely stems from the relatively small number of cases and uneven case distribution within that interval in our retrospective cohort, causing minor fluctuations in the fitted calibration curve. Crucially, comparison of diagnostic efficacy revealed that the nomogram model achieved a significantly superior AUC compared to a model based solely on conventional clinical indicators. This robust finding further substantiates the diagnostic superiority of the integrated model incorporating both MRI parameters and molecular biomarkers for PCa. Collectively, these results indicate that the combined diagnostic model offers significantly enhanced accuracy in predicting PCa risk, thereby providing substantial support for clinical decision-making regarding diagnosis and therapeutic strategy selection. Compared with current diagnostic practice, this model may help reduce unnecessary prostate biopsies by more accurately distinguishing clinically significant PCa from benign or indolent lesions. Moreover, the integration of imaging and biomarker data could enhance patient risk stratification and assist clinicians in making more individualized management decisions. Although the addition of biomarker testing may increase initial diagnostic costs, it could ultimately prove cost-effective by decreasing unnecessary biopsies and reducing procedure-related morbidity.

In summary, this study successfully developed and validated a combined diagnostic model for PCa by integrating MRI parameters with key gene expression biomarkers. The results affirm that this model possesses high predictive accuracy and considerable potential for clinical application. Nevertheless, certain limitations warrant acknowledgment. The retrospective, single-center design introduces possible selection bias. Therefore, future studies with external, multi-center validation are warranted to confirm its generalizability. Future prospective, multi-center studies are planned to externally validate the nomogram and further assess its robustness in diverse patient populations. In addition, the semi-quantitative evaluation of p504s, CK5/6, and Ki-67 was operator-dependent, which may introduce subjectivity and affect reproducibility. Future incorporation of digital or AI-based image analysis could provide more objective and standardized quantification of these biomarkers. Also, the current study did not include a decision curve comparison between the MRI-only and nomogram models due to dataset constraints. Future research will aim to incorporate such analyses to further validate the incremental clinical utility of the combined model.

## References

[1] Achard V, Putora PM, Omlin A, Zilli T, Fischer S (2022). Metastatic prostate cancer: treatment options. Oncology.

[2] Aina T, Salifu AA, Kizhakkepura S, Danyuo Y, Obayemi JD, Oparah JC (2023). Sustained release of alpha-methylacyl-CoA racemase (AMACR) antibody-conjugated and free doxorubicin from silica nanoparticles for prostate cancer cell growth inhibition. J Biomed Mater Res B Appl Biomater.

[3] Hugosson J, Godtman RA, Wallstrom J, Axcrona U, Bergh A, Egevad L (2024). Results after four years of screening for prostate cancer with PSA and MRI. N Engl J Med.

[4] Lewicki P, Morgan T (2024). MRI-visible and -invisible prostate cancer. J Urol.

[5] Caglic I, Sushentsev N, Syer T, Lee KL, Barrett T (2024). Biparametric MRI in prostate cancer during active surveillance: is it safe?. Eur Radiol.

[6] Johnson PM, Chandarana H (2024). AI-powered diagnostics: transforming prostate cancer diagnosis with MRI. Radiology.

[7] Völkel C, De Wispelaere N, Weidemann S, Gorbokon N, Lennartz M, Luebke AM (2022). Cytokeratin 5 and cytokeratin 6 expressions are unconnected in normal and cancerous tissues and have separate diagnostic implications. Virchows Arch.

[8] Blessin NC, Yang C, Mandelkow T, Raedler JB, Li W, Bady E (2023). Automated Ki-67 labeling index assessment in prostate cancer using artificial intelligence and multiplex fluorescence immunohistochemistry. J Pathol.

[9] Zheng L, Ling W, Zhu D, Li Z, Li Y, Zhou H (2023). Roquin-1 resolves sepsis-associated acute liver injury by regulating inflammatory profiles via miRNA cargo in extracellular vesicles. iScience.

[10] Kulac I, Roudier MP, Haffner MC (2024). Molecular pathology of prostate cancer. Clin Lab Med.

[11] Wilson TK, Zishiri OT (2024). Prostate cancer: a review of genetics, current biomarkers and personalised treatments. Cancer Rep Hob.

[12] Fazekas T, Shim SR, Basile G, Baboudjian M, Kói T, Przydacz M (2024). Magnetic resonance imaging in prostate cancer screening: a systematic review and meta-analysis. JAMA Oncol.

[13] Yoshizawa A, Takahara K, Saruta M, Zennami K, Nukaya T, Fukaya K (2021). Combined α-methylacyl-CoA racemase inhibition and docetaxel treatment reduce cell proliferation and decrease expression of heat shock protein 27 in androgen receptor-variant-7-positive prostate cancer cells. Prostate Int.

[14] Fang AM, Gregg JR, Pettaway C, Ma J, Szklaruk J, Bathala TK (2025). Whole-body MRI for staging prostate cancer: a narrative review. BJU Int.

[15] Iakymenko OA, Briski LM, Delma KS, Jorda M, Kryvenko ON (2022). Utility of D2-40, cytokeratin 5/6, and high-molecular-weight cytokeratin (clone 34βE12) in distinguishing intraductal spread of urothelial carcinoma from prostatic stromal invasion. Am J Surg Pathol.

[16] Song Z, Zhou Q, Zhang JL, Ouyang J, Zhang ZY (2024). Marker Ki-67 is a potential biomarker for the diagnosis and prognosis of prostate cancer based on two cohorts. World J Clin Cases.

[17] Wang Y, Wang L, Tang X, Zhang Y, Zhang N, Zhi B (2023). Development and validation of a nomogram based on biparametric MRI PI-RADS v2.1 and clinical parameters to avoid unnecessary prostate biopsies. BMC Med Imaging.

[18] Cavallo AU, Stanzione A, Ponsiglione A, Trotta R, Fanni SC, Ghezzo S (2025). Prostate cancer MRI methodological radiomics score: a EuSoMII radiomics auditing group initiative. Eur Radiol.

[19] Van der Kwast TH (2023). Proliferative cribriform prostate cancer: a new opportunity for ’promising’ marker KI-67?. Histopathology.

[20] Lima H, Etchebehere M, Bogoni M, Torricelli C, Nogueira-Lima E, Deflon VM (2024). Theranostics nuclear medicine in prostate cancer. Pharmaceuticals.

[21] Faur IF, Dobrescu A, Clim IA, Pasca P, Prodan-Barbulescu C, Tarta C (2024). The predictive role of serum lipid levels, p53 and ki-67, according to molecular subtypes in breast cancer: a randomized clinical study. Int J Mol Sci.

[22] Jha N, Phulware RH, Kumar A, Singh A, Durgapal P, Chowdhury N (2024). A study of Ki-67 immunostaining in prostate carcinomas and its correlation with gleason’s score and prognosis: an experience at a tertiary centre in the himalayan foothills. Indian J Surg Oncol.

